# Oral Gel Loaded by Fluconazole‒Sesame Oil Nanotransfersomes: Development, Optimization, and Assessment of Antifungal Activity

**DOI:** 10.3390/pharmaceutics13010027

**Published:** 2020-12-25

**Authors:** Hala M. Alkhalidi, Khaled M. Hosny, Waleed Y. Rizg

**Affiliations:** 1 Department of Clinical Pharmacy, Faculty of Pharmacy, King Abdulaziz University, Jeddah 21589, Saudi Arabia; halkhaldi@kau.edu.sa; 2 Department of Pharmaceutics, Faculty of Pharmacy, King Abdulaziz University, Jeddah 21589, Saudi Arabia; wrizq@kau.edu.sa; 3 Center of Excellence for Drug Research and Pharmaceutical Industries, King Abdulaziz University, Jeddah 21589, Saudi Arabia

**Keywords:** fluconazole, Box‒Behnken design, nanotransfersome, ulcer index, zone of inhibition, rheological behavior, ex vivo permeation

## Abstract

Candidiasis is one of the frequently encountered opportunistic infections in the oral cavity and can be found in acute and chronic presentations. The study aimed to develop fluconazole-loaded sesame oil containing nanotransfersomes (FS-NTF) by the thin-layer evaporation technique to improve the local treatment of oral candidiasis. Optimization of the formulation was performed using the Box‒Behnken statistical design to determine the variable parameters that influence the vesicle size, entrapment efficiency, zone of inhibition, and ulcer index. Finally, the formulated FS-NTF was embedded within the hyaluronic acid‒based hydrogel (HA-FS-NTF). The rheological behavior of the optimized HA-FS-NTF was assessed and the thixotropic behavior with the pseudoplastic flow was recorded; this is desirable for an oral application. An in vitro release study revealed the rapid release of fluconazole from the HA-FS-NTF. This was significantly higher when compared with the fluconazole suspension and hyaluronic acid hydrogel containing fluconazole. Correspondingly, the ex vivo permeation was also found to be higher in HA-FS-NTF in sheep buccal mucosa (400 μg/cm^2^) when compared with the fluconazole suspension (122 μg/cm^2^) and hyaluronic acid hydrogel (294 μg/cm^2^). The optimized formulation had an inhibition zone of 14.33 ± 0.76 mm and enhanced antifungal efficacy for the ulcer index (0.67 ± 0.29) in immunocompromised animals with *Candida* infection; these findings were superior to those of other tested formulations. Hence, it can be summarized that fluconazole can effectively be delivered for the treatment of oral candidiasis when it is entrapped in a nanotransfersome carrier and embedded into cross-linked hyaluronic acid hydrogel.

## 1. Introduction

Oral candidiasis is a frequently encountered infection of the oral mucosa. It can be caused by the overgrowth of 150 species of *Candida*; however, 95% cases are caused by *Candida* albicans [1,2]. This is sometimes mild in nature, but may be resistant to therapies and is frequently vulnerable to relapse.

The drug of choice for the treatment of oral candidiasis is fluconazole, a fluorinated bis-triazole derivative. This agent has been found to be effective against several species of *Candida* in both immunocompromised and immunocompetent patients [3]. The mechanism of inhibiting *Candida* spp. infection involves the inhibitory effect of 14-α-demethylase, which is required for ergosterol biosynthesis and thereby interrupts the cell wall synthesis of the fungi. The common route of administration of conventional fluconazole is oral; however, this route is known to produce gastrointestinal disturbances, such as abdominal discomfort, bloating, vomiting, and severe hepatotoxicity [4]. Localized delivery of the medication for the treatment of oral candidiasis could reduce the development of drug resistance and also reduce the associated side effects [3]. Conventional local forms of delivery of fluconazole (e.g., sprays, lotions, gels, and creams) are associated with major limitations in dosing accuracy and length of time at the site of application, as well as variations in performance. Lozenges, troches, rinses, and mouth paints are the alternate treatment options for oral candidiasis; however, maintenance of the salivary concentration of the medication is difficult because salivary secretions can wash away these substances and shorten the contact time of the formulation, leading to poor efficacy and poor patient compliance [4,5]. Therefore, novel formulations with properties that are retained at the site of application and release the entrapped drug for a desired period would be a possible alternative to conventional deliveries. Researchers have used various approaches to deliver fluconazole effectively and with improved efficacy. They used mucoadhesive platforms, such as fluconazole-loaded oral strips [3], mucoadhesive nanoparticles [4], hydrogels [6], and natural rubber latex biomembranes [7], among others.

In view of the advantages of longer retainment at the site of application for the mucoadhesive components and the superior release characteristics of the nanotechnology-based products, the present study aimed to formulate fluconazole-loaded sesame oil containing nanotransfersomes (FS-NTF) embedded in a cross-linked hyaluronic acid hydrogel. A new field of research on topical delivery was opened with the use of liposomes for dermal delivery, and since then, a wide range of novel lipid-based vesicles, such as deformable liposomes in nanosizes, which are currently known as nanotransfersomes, have been developed. The addition of nonionic surfactants to the liposomal bilayer structure of liposomes provides the flexibility necessary for liposomes, and this new structure is called the nanotransfersome [8]. In the proliferation phase, in which granulation tissue is formed, hyaluronic acid is synthesized mainly by fibroblasts, and this allows, within the framework of a temporary extracellular matrix, the diffusion of nutrients and the elimination of waste products. Hyaluronic acid facilitates the migration and proliferation of fibroblasts and keratinocytes and is a reservoir of growth factors. This is due to the ability of hyaluronic acid to absorb water, maintain wound moisture, and limit cellular adhesion to extracellular matrix molecules [9]. Hyaluronic acid has also been shown to possess antifungal efficacy, especially fungistatic efficacy [10]. Therefore, it was expected that superior efficacy would be achieved by the FS-NTF embedded in hyaluronic acid (HA-FS-NTF). It was expected that the formulation would remain at the site of application because of the swelling properties of hyaluronic acid [11]. Additionally, incorporation of sesame oil in the formulation would be advantageous for its established antifungal efficacy [12]. To reach this goal, the optimization of the FS-NTF was performed by the Box‒Behnken statistical design to determine the various parameters that influence the vesicle size, entrapment efficiency, zone of inhibition, and ulcer index. Finally, the HA-FS-NTF was formulated and evaluated for its rheological behavior, in vitro release pattern, ex vivo permeability, inhibitory zone of *Candida* growth, and ulcer index in an immunocompromised animal model.

## 2. Materials and Methods

### 2.1. Materials

Fluconazole was purchased from Sigma-Aldrich Co. (St Louis, MO, USA). Tween 20^®^ was acquired as a generous gift from Saudi Drugs and Medical Instruments Company (SPIMACO), in Qassim, Saudi Arabia. Sesame oil and Lecithin were procured from Avanti Polar Liquids (Alabaster, AL, USA). High-performance liquid chromatography (HPLC)-grade solvents were collected form Merck (Darmstadt, Germany). All other reagents and chemicals used were of analytical grade.

### 2.2. Methods

#### 2.2.1. Optimization of Fluconazole‒Sesame Oil Nanotransfersomes Using the Box–Behnken Statistical Design

A response surface Box–Behnken statistical design was adopted for our current investigation using Design-Expert^®^ software version 12.0.6.0 (2019, Stat-Ease, Inc., Minneapolis, MN, USA). Three factors at three levels were incorporated to optimize the fluconazole nanotransfersome formulations. Based on data in the literature, higher and lower levels of the independent variables, such as the lecithin concentration and amounts of fluconazole and sesame oil, were selected for the identification of the optimized FS-NTF formulation [10,13]. The software generated 19 batches with various combinations of the three independent variables at their low (−1), medium (0), and high (+1) levels. The batches were developed and analyzed for the four dependent variables, which were the globule size of the prepared FS-NTF (Y1), entrapment efficiency (EE%) of fluconazole within the prepared FS-NTF (Y2), zone of inhibition against *C. albicans* (Y3), and ulcer index score (Y4) (Table 1). Experimental data for the four dependent variables were included in the software. The interactions of the independent variables at their different levels with the dependent variables were analyzed to gain insight into the composition of the optimized formulation. The significance of the data was statistically analyzed using the analysis of variance (ANOVA). The effect of the three independent variables at their different levels was assessed by the generated perturbation plot, contour plots, and three-dimensional surface plot [14,15]. The best fitting model was used based on data on the adequate precision ratio and the predicted and adjusted determination coefficients for the dependent variables.

The software generated a polynomial equation consisting of three factors, and the responses are depicted in the following Equation (1):

Y = b0 + b1A + b2B + b3C + b12AB + b13AC + b23BC + b11A2 + b22B2 + b33C2(1)

where Y was the measured response associated with each factor level combination, b0 was the intercept, b1 to b3 were the regression coefficients [16], and A, B, and C were the coded levels of the independent variables.

#### 2.2.2. Preparation of Fluconazole‒Sesame Oil Nanotransfersomes (FS-NTF)

**Figure 1:**
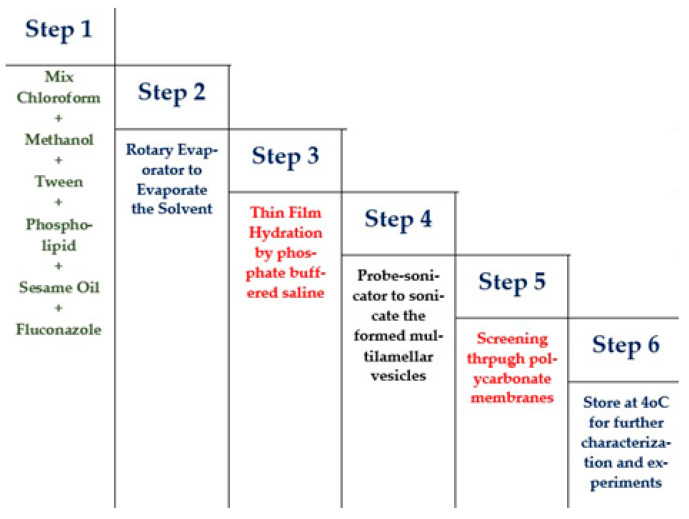
Schematic representation of steps of preparation of fluconazole‒sesame oil nanotransfersomes (FS-NTF).

Development of the drug-loaded NTF was approached by the thin-layer evaporation technique using the optimized ratios of the components suggested by the statistical design [17]. In brief, accurately measured quantities of Tween 20^®^, as an edge activator, were incorporated with the phospholipid to form the vesicular structure of the nanotransfersome. The Tween^®^ and phospholipid were placed in a round-bottom flask, along with the sesame oil and fluconazole, using the solvent mixture of chloroform:methanol at a ratio of 1:1 (*v*/*v*). The organic solvents in the round-bottom flask were evaporated at reduced pressure using the rotary vacuum evaporator (BUCHI Rotavapor R-205, Marshall Scientific, Hampton, NH, USA) at 45 °C, and the rotation of the flask was maintained at 80 rpm. Later, following the removal of traces of organic solvents in the mixture, a thin film of the components was formed within the inner wall of the round-bottom flask. Lastly, phosphate buffered saline (pH 7.4) was used to hydrate the thin layer in order to form the lipid vesicles at room temperature. The multilamellar vesicles were formed upon hydration, and they were further sonicated using a probe sonicator (Branson, Danbury, CT, USA) for 15 min to form our desired drug-loaded unilamellar vesicles (FS-NTF). Finally, the formulation was collected after it had passed through polycarbonate membranes of the desired size and stored at 4 °C for further characterization and experiments. Figure 1

#### 2.2.3. Characterization of the Fluconazole-Loaded Nanotransfersomes

##### Globule Size Measurements

The vesicle size of the developed FS-NTF was measured by appropriately diluting the mixture with purified water (10 times), and the mixture was agitated vigorously before measurement. The size of the formulated vesicles was finally determined by the dynamic light-scattering technique, in which 100 µL of the diluted sample was placed in the Microtrac^®^ particle size analyzer (Microtrac, Inc., Montgomeryville, PA, USA) at 25 ± 1 °C. The average readings were recorded to correlate with our findings.

##### Entrapment Efficiency Percentage (EE%)

The EE% of the developed and optimized FS-NTF was determined using the indirect method [18], where the EE% was stated as the percentage of the total amount of drugs incorporated within the final formulation. To perform this experiment, the FS-NTF was freeze dried (Martin Christ Gefriertrocknungsanlagen GmbH, Osterode am Harz, Germany) first to remove the traces of the aqueous phase in the formulation. Later, it was dispersed with methanol and mixed cautiously. The mixture was centrifuged (Centurion, West Sussex, UK), and the supernatant was collected. The residue was washed again with the same solvent to recover the traces of the free fluconazole present in the developed formulation. The supernatant containing the drug was measured to determine the concentration of free fluconazole in the formulation using a UV-visible spectrophotometer (6705 UV/Vis spectrophotometer; JENWAY, Cole-Parmer, Stone, Staffordshire, UK) at 261.6 nm. The correlation coefficient was 0.9997, and the molar absorptivity equaled 0.735 × 10^3^ L/mol/cm [19].

Finally, the EE% of the formulation was determined using Equation (2).

(2) $$EE\%=\frac{C_{total}-C_{free}}{C_{total}}\times 100$$

where *C_total_* and *C_free_* are the total quantity of drug added in each formulation according to the experimental design mentioned in Table 1 (25, 50, or 75 mg) and the free drug available outside the formed nanotransfersome vesicles, respectively.

##### Development of Hyaluronic Acid Hydrogel of FS-NTF (HA-FS-NTF)

The formulated FS-NTF was embedded into the hyaluronic acid hydrogel by sprinkling it on the pH-induced hyaluronic acid hydrogel. This polymeric hydrogel was prepared with 2% hyaluronic acid using 0.5% Gantrez^®^ S-97 (Mw = 1.2 × 10^6^ Da) as a cross-linker in a phosphate buffer saline (PBS) media (pH 4.5) maintained at 37 ± 1 °C according to preliminary tests which were performed to determine the optimum levels for hyaluronic acid and cross linker. The polymeric mixture was left overnight to hydrate with continuous stirring at 110 rpm. Finally, the HA-FS-NTF was developed by mixing 10 mL of the formulated FS-NTF containing 600 mg of the drug into the prepared hydrogel (100 mL) to obtain the final 0.5% drug containing the transfersomal hydrogel preparation. The FS-NTF was dispersed in water, and this aqueous dispersion was sprinkled over the plain hydrogel base. Because hyaluronic acid has a higher affinity to absorb water molecules and swell, the state of the water in swollen hydrogels is an important factor that influences the absorption and diffusion of solutes through the hydrogel. Generally, water consists of bound water and free water in water-swollen systems. When a hydrogel network begins to swell, the hydrophilic and hydrophobic groups of polymer chains interact with the water molecules, leading to “bound water.” Then the network will absorb additional water, due to the osmotic pressure, leading to “free water” that fills the space between the network chains. This HA-FS-NTF was refrigerated at 4 °C to 8 °C and tested for content uniformity by dividing a certain amount of the formed gel into pieces of equal weight and analyzing these pieces for fluconazole content. After ensuring the content uniformity, further evaluation was done.

#### 2.2.4. Rheological Evaluation of the HA-FS-NTF Hydrogel

The rheological property, viscosity, of the HA-FS-NTF and the plain hyaluronic acid gel was measured using a Brookfield Viscometer fitted with a spindle 52. A quantity equivalent to 1 g of the swollen tested hydrogel sample was used to determine the desired parameters, and the run of the spindle was performed at 25 ± 5 °C [20]. The findings were recorded over a range of shear rates (2, 10, 20, 30, 40, 50, and 60 s^−1^) to identify the flow pattern of the formulated gels. Thus, the shear rate (s^−1^), shear stress (dyne/cm^2^), and viscosity (cP) were recorded and plotted. The viscosity and degree of pseudoplasticity (Farrow’s constant) were determined at a minimum rate of shear (ηmin) and a maximum rate of shear (ηmax) by Farrow’s equation (Equation (3)) [21].

LogG = N LogF − Log ղ(3)

where the shear rate, viscosity, and shear stress are expressed as G, ղ, and F, respectively, and Farrow’s constant is expressed as N.

#### 2.2.5. In Vitro Release of the FS-NTF-Loaded Hydrogel

In vitro release of the entrapped drug from the developed HA-FS-NTF containing the 0.5% marketed formulation of fluconazole and the suspension containing 0.5% fluconazole was studied using a Type I USP dissolution apparatus (basket type) (DT 700 LH device, ERWEKA GmbH DT 700, Heusenstamm, Germany). The samples were loaded into the respective cylindrical tubes (10 cm in length and 2.7 cm in diameter) and were attached to the apparatus instead of the basket, with the lower end of the tube closed tightly by a semipermeable membrane with a pore size of 100 µm. Before use, the dialysis membrane was activated by placing the dialysis membrane in boiling water containing 1 M NaHCO_3_ for 1 h. Thereafter, it was washed thoroughly using tap water and stored in phosphate buffer (pH 7.4) for 1 h. The dialysis membranes were kept in the refrigerator in the phosphate buffer (pH 7.4) media overnight prior to performing the release study.

One gram of each sample was loaded in the cylindrical glass tube assembly as described above. The release study was performed in 250 mL phosphate buffered saline (pH 6.8), which was maintained at a physiological temperature (37 ± 0.5 °C) with the rotation of the stirring shaft fixed at 50 rpm. The release study was performed for 3 h, during which time 5-mL aliquots were withdrawn at intervals of 0.0, 0.25, 0.5, 1.0, 1.5, 2.0, 2.5, and 3.0 h. Fresh media were replaced during each withdrawal and maintained at room temperature. The collected samples were filtered using a 0.45-m membrane filter, and the rate of drug release was determined using a UV-visible spectrophotometer at 261.6 nm, with phosphate buffered saline as the blank.

#### 2.2.6. Ex Vivo Skin Permeation Studies

An ex vivo permeability study was performed using sheep buccal mucous membrane by the Franz diffusion cell method (MicroettePlus^®^, Teledyne Hanson, Chatsworth, CA, USA). The freshly excised buccal mucous membrane was obtained from the slaughterhouse and kept in phosphate buffered saline. A prepared buccal mucous membrane of 2 × 2 cm was carefully arranged between the acceptor and donor compartments of the diffusion cell area (1.75 cm^2^). The permeation characteristics were evaluated for the developed HA-FS-NTF containing the 0.5% marketed formulation of fluconazole and the suspension containing the 0.5% fluconazole. Eight milliliters of phosphate buffered saline (pH 6.8) prepared from disodium hydrogen phosphate (Na_2_HPO_4_ anhydrous) = 14.4 g sodium dihydrogen phosphate (KH_2_PO_4_ anhydrous) = 2.4 g sodium chloride (NaCl) = 80.0 g, and potassium chloride (KCl) = 2.0 g, was kept in the receptor chamber, which was maintained at 32 ± 2 °C with the help of a warm water jacket and stirred at 410 to 430 rpm using the magnetic bead. The samples were autosampled at predetermined time intervals and quantified in parallel using high-performance liquid chromatography (HPLC) [22]. Diffusion of the fluconazole from the donor compartment to the receptor compartment through the mounted mucous membrane was determined by plotting the cumulative release pattern of the drug per unit of time and area. We calculated the diffusion coefficient (D), enhancement factor (EF), permeability coefficient (Pc), and steady-state flux (Jss) to determine whether the developed formulation was superior. Finally, the percentage of fluconazole permeated and the total amount of fluconazole diffused across the receptor chamber was calculated using Equation (4):

(4) $$Percent\;permeated=\frac{F_{P}}{F_{T}}\times 100$$

where *Fp* and *F_T_* are the permeated amount of fluconazole in the receptor chamber and the loaded amount of fluconazole in the donor chamber, respectively.

#### 2.2.7. Determination of Zone of Inhibition against *Candida* *Albicans*

The antifungal efficacy of the formulated formulation was determined by the disk diffusion method. We used the Sabouraud dextrose agar medium to grow *C. albicans* strains for inoculum preparation and the conduction of in vitro antifungal evaluation using the fungal strains.

##### Preparation of Inoculum

Four to five colonies of standard *C. albicans* strains were selected and suspended in normal saline (2 mL) and mixed vigorously. The turbidity of the fungal suspension was maintained homogeneously by the use of the 0.6-meq McFarland standards. Thereafter, streaks were made on the Sabouraud dextrose agar plate by dipping a sterile swab into the prepared fungal suspension to get the lawn culture.

##### Disk Diffusion Method

Sterile filter paper discs of 5 mm in diameter were soaked with 100 μL of the FS-NTF and ethanol (99.9%) as test and positive control samples, respectively. The soaked paper disks were placed on inoculated Sabouraud dextrose agar plates. The inoculated plates were incubated at 24 ± 2 °C for 48 h and then examined for their respective zones of inhibition.

#### 2.2.8. Determination of Ulcer Index

##### Animals

Male albino rats weighing between 160 and 240 g were selected for the in vivo determination of the ulcer index. The experimental animals were procured from the animal house facility of the Clinical Laboratory Center, Beni Suef, Egypt. The animals were housed in standard laboratory conditions (temperature, 25 ± 2 °C; relative humidity, 55 ± 5%) with free access to water and food for 7 days [16]. Thereafter, the animals were used for the current experiment following approval of the protocol by the Institutional Review Board for Animal Research/Studies Animals (Approval No.15-08-2020).

##### Preparation of Organisms for Inoculation in Animal Model

Four to five colonies of standard *C. albicans* strains were selected and suspended in normal saline (2 mL) and mixed vigorously. The suspended cells were collected by centrifugation at 2500× *g* for 10 min. Thereafter, the pelleted cells were washed with phosphate buffered saline thrice and finally suspended in the buffer to obtain the final cell concentration of 3 × 10^8^ colony-forming units (CFU)/mL.

##### Formation of Oral Candidiasis in the Rat Model and Evaluation of the Developed Formulation

##### Preparation of Immunocompromised Model

The efficacy of the developed HA-FS-NTZ for the oral candidiasis model was evaluated in immunocompromised animal models following the protocol of Martinez and team [23]. The acclimatized animals were immunocompromised first by the consumption of dexamethasone with tetracycline. The initial dose of dexamethasone (0.5 mg/L) and tetracycline (1 g/L) was supplied to the animals via drinking water for 7 days, followed by an increase in the concentration of dexamethasone (1 mg/L) and a reduction in the concentration of tetracycline (0.1 g/L) 1 day before infecting the animals. The same dose of dexamethasone with tetracycline was continued until the end of the study.

##### Inoculation of *C. albicans* Strains to the Immunocompromised Animals

The oral cavities of the experimental animals were checked for infection with *C. albicans*. Following the confirmed absence of *C. albicans* infections, the animals were orally infected by rolling a cotton swab containing 0.1 mL of the prepared fungal suspension thrice over the entire oral cavity on days 3, 5, and 7. Confirmation of infection in the experimental animals and quantification of the number of CFUs in the oral cavity were performed by an oral swab test after 72 h from the last inoculation.

##### Treatment of the Infected Animals

The animals were divided into six groups. The control group was treated with normal saline, and the animals in the other five groups were treated with formulated HA-FS-NTF, hyaluronic acid hydrogel loaded with F-NTF (without sesame oil), hyaluronic acid hydrogel loaded with fluconazole, fluconazole aqueous suspension, and blank hyaluronic acid hydrogel. The treatments were continued for 3 consecutive days, and the ulcer indexes were determined on third day [24,25]. Scoring of the oral ulcer index in the experimental animals was measured as shown in Table 2.

#### 2.2.9. Statistical Analysis

All of the presented results were expressed as the mean ± the standard deviation. The comparisons of results between the different groups were performed statistically using the paired *t*-test, where *p* < 0.05 was considered a significant difference between the compared groups.

## 3. Results and Discussion

### 3.1. Formulation of Fluconazole-Loaded Nanotransfersome

The edge activators in the nanotransfersome formulations played a definitive role. They make the formed vesicles ultraflexible so that they are capable of noninvasively penetrating the skin by virtue of their high level of self-optimizing deformability. An elastic nanotransfersomal carrier is described as a lipid droplet of such deformability that it permits easy penetration through pores much smaller than the droplet size, and, also, it is a highly adaptable and stress-responsive complex aggregate. The vesicles are elastic and very deformable; they consist of lecithin in combination with an edge-active surfactant such as Tween. An edge activator is usually a single-chain surfactant that causes the destabilization of the lipid bilayer of the vesicle and increases the vesicle elasticity or fluidity by lowering its interfacial tension. Amongst the various edge activators used widely by different researchers, Spans, Tweens, potassium glycyrrhizinate, sodium deoxycholate, and sodium cholate are widely incorporated for the development of stable and effective formulations [17]. The current approach of formulating fluconazole-loaded nanotransfersomes was achieved by incorporating Tween 20. Tween 20 is a nonionic polyoxyethylene sorbitol ester widely used in pharmaceutical preparations due to its biocompatible characteristics. The development of the nanotransfersomes progressed using this nonionic surfactant [26].

We also incorporated sesame oil in the nanotransfersome formulation. Sesame oil is traditionally used as a pulling agent for dentures; it prevents malodor, bleeding from the gums, tooth decay, and dryness of the lips and throat [27]. Further studies revealed a concentration-dependent antifungal role of sesame oil, specifically against *C. albicans* [10]. Therefore, a total of 19 formulations were prepared according to the Design of Experiment model to formulate an optimized formulation for the next stage of experiment.

### 3.2. Optimization of the Fluconazole‒Sesame Oil Nanotransfersome: Box–Behnken Statistical Design

#### 3.2.1. Optimization of the Globule Size of the Fluconazole‒Sesame Oil Nanotransfersome Formulations

The globule size of a nanotransfersome formulation is one important parameter in formulation optimization because it can affect the solubility and drug permeation through the skin [28]. Therefore, we analyzed the interactions of the three independent variables in our formulation to determine the globule size of the FS-NTF. During the optimization process, the quadratic model was found to be the best fit. Table 3 shows the statistical outcome of the interactions between the amount of lecithin, fluconazole, and sesame oil in the formulation and the globule size of the FS-NTF. The statistical significance of the different model terms is represented by the *p*-value (<0.05). The model’s F-value of 321.4 represents the significance of the model. A lack-of-fit F-value of 4.88 and a respective *p*-value (>0.05) indicated that the lack of fit was not significant relative to the pure error, and this was desirable because we wanted the model to be fit. Close agreement was observed between the actual and predicted globule sizes of the formulations, as shown in Table 1, and also in the predicted and adjusted R^2^ values, with a difference of less than 0.2 for the dependent variable of globule size.

The model terms A, B, C, AB, and A2 were significant; they represent the significant effects of the amounts of lecithin, fluconazole, and sesame oil on the globule size of the formulations. A desirable signal-to-noise ratio is higher than 4, and the suggested model has a value of 55.071, indicating an adequate signal. Therefore, this model could be used to navigate the design space.

The generated quadratic equation on the interactions of the three independent variables with the globule size of the formulations is shown in Equation (5).

Y1 = +219.67 + 51.40*A + 37.95*B − 6.07*C − 29.56*A^2^ + 11.05*A*B + 1.30*A*C + 1.02*B^2^ + 0.0489*B*C − 1.63*C^2^ (5)

**Figure 2:**
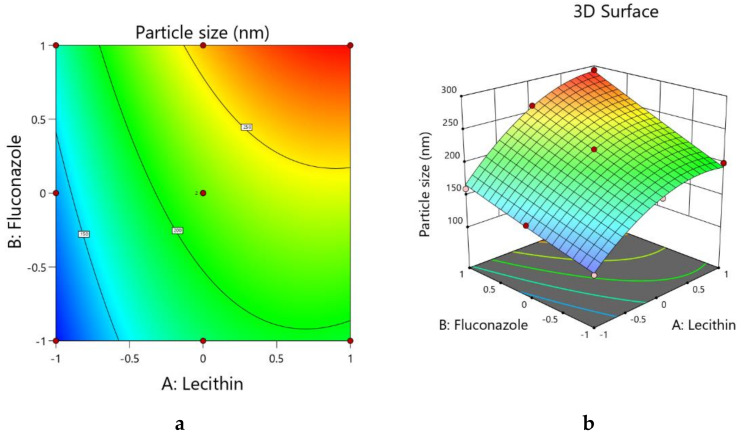
(**a**) Contour plot showing the interactions of lecithin and fluconazole with the globule size of the FS-NTF. (**b**) 3D surface plot showing the interactions of lecithin and fluconazole with the globule size of the FS-NTF.

Model terms A and B have positive coefficient values of +51.40 and +37.95, respectively, whereas model term C has a negative coefficient value (−6.07) (Equation (5)). The *p*-value for all the model terms was less than 0.05 (Table 3); this indicates a significant increase in the particle size with increasing amounts of lecithin and fluconazole and a decreasing amount of sesame oil in the formulation. The higher value of coefficient of model term A indicates a higher effect on the particle size with changes in the amount of lecithin, followed by the amounts of fluconazole and sesame oil in the formulations. Lecithin is a phospholipid that is responsible for the formation of the concentric lipid bilayers of the formed vesicles. This lipid bilayer is the medium in which the lipophilic drug is located. For this reason, the increase in the lecithin concentration within the formulated liposomes leads to an increased number of bilayers of this multilamellar vesicle, and this leads to an increase in the size of the formed vesicles. A similar effect is reflected in the perturbation plot, where positive slopes are evident with model terms A and B and there is a flattened negative curve with model term C. The curve with model term A is stiffer than the curve with model term B, and this is per the respective coefficient value in Equation (2). Similar effects on the interaction of the amounts of lecithin and fluconazole are reflected by color changes in the contour plot (see Figure 2a) and by changes in the color and slope of the three-dimensional surface plot (see Figure 2b).

#### 3.2.2. Box–Behnken Statistical Designs: Optimization of the Entrapment Efficiency of the Fluconazole‒Sesame Oil Nanotransfersome Formulations

The EE% of the nanoformulation is one of the hurdles in developing novel deliveries, where nanoformulations with a higher EE% are desirable [29]. Therefore, we analyzed the interactions of the three independent variables in our formulation to achieve a higher percentage of fluconazole to be successfully entrapped in the FS-NTF. During the optimization process, the quadratic model was found to be the best fit for a response such as the EE%. The statistical outcome of the interactions of the amounts of lecithin, fluconazole, and sesame oil in the formulation on the EE% of the FS-NTF is shown in Table 4. The model *F*-value of 386.29 represents the significance of the model. An F-value of 0.76 for the lack of fit and the respective *p*-value (>0.05) indicated that the lack of fit was not significant relative to the pure error; this was desirable as it indicated the model was fit. Close agreement was observed between the predicted and actual EE% of the formulations, as shown in Table 1, and the difference between the predicted and actual R^2^ values was found to be less than 0.2.

In Table 4, the *p*-value indicates a significant effect of the amounts of lecithin, fluconazole, and sesame oil on the EE% of the formulations. When the coefficient value of model terms A, B, and C in the quadratic equation (Equation (6)) indicated that a desirable signal-to-noise ratio was more than 4, we found that the suggested model had a value of 60.699, indicating an adequate signal. Therefore, this model could be used to navigate the design space.

The generated quadratic equation on the interactions of the three independent variables on the EE% of the formulations is shown in Equation (6).

Y2 = +75.19 + 3.70*A − 3.10*B + 1.38*C − 0.9091*A^2^ − 0.1381*A*B − 0.3881*A*C − 16.57*B^2^ − 0.1381*B*C − 0.2650*C^2^ (6)

**Figure 3:**
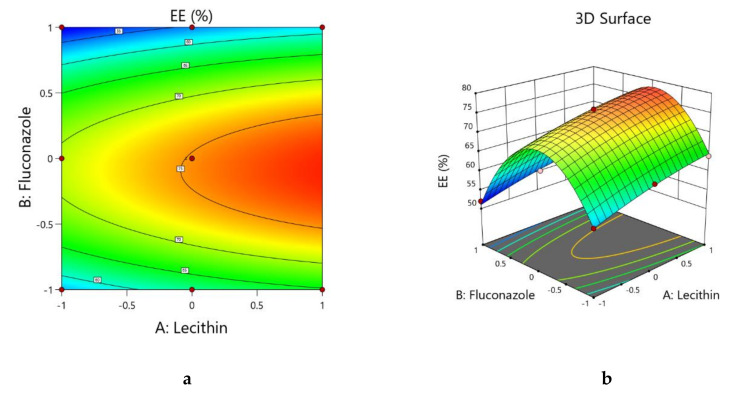
(**a**) Contour plot showing the interactions of lecithin and fluconazole with the EE% of the FS-NTF. (**b**) Three-dimensional surface plot showing the interactions of lecithin and fluconazole with the EE% of the FS-NTF.

From the equation, it can be seen that the model terms A and C have a positive coefficient value of +3.70 and +1.38, respectively, whereas model term B has a negative coefficient value (−3.10). The p-value for all the model terms was less than 0.05 (Table 4). This indicates a significant increase in the EE% with an increasing amount of lecithin and sesame oil and a decreasing amount of fluconazole in the formulation. A similar effect is reflected in the perturbation plot, where positive slopes are evident with model terms A and C and a positive slope is observed for model term C up to a certain extent and the curve of the model term goes down with an increasing amount of fluconazole. The curve with model term A is stiffer than the curve with model term C; this is per the respective coefficient value in Equation (3), so it can be inferred that the effect of the amount of lecithin is higher than for the amount of sesame oil in the EE% of the formulation. The effect of the interactions between the amounts of lecithin and fluconazole is reflected by the color changes in the contour plot (Figure 3a) and by the changes in color and slope in the three-dimensional surface plot (see Figure 3b). It is interesting to note that for the contour plot and three-dimensional surface plot (see Figure 3), an increasing amount of drug in the nanotransfersome formulation leads to an increase in the EE% up to a certain point; however, a further increase in drug concentration leads to a decrease in the EE% of the formulation. This phenomenon might be explained by the inefficiency of entrapping a higher amount of drug in the nanotransfersome formulation. At each level for the lecithin amount, the increase of fluconazole will increase the EE% until a certain level is reached, but then a further increase in the level of fluconazole will lead to a decrease in the EE%. This occurs as the space available for the incorporation of the added fluconazole becomes insufficient for the excess amount added, consequently leading to a decrease in the EE% at a higher level of fluconazole.

#### 3.2.3. Box–Behnken Statistical Designs: Optimization of Zone of Inhibition of Fluconazole‒Sesame Oil Nanotransfersome Formulations

To assess the antifungal activity, we analyzed the interactions of the three independent variables of the zone of inhibition of the FS-NTF. During the optimization process, it was observed that the quadratic model was the best fit for the zone of inhibition of the formulation. The statistical outcome of the interactions of the amounts of lecithin, fluconazole, and sesame oil in the formulation’s zone of inhibition of the FS-NTF is shown in Table 5. The model’s F-value is 818.62, and the respective *p*-value of less than 0.05 represents the significance of the model. An F-value of 0.4747 for the lack of fit and respective *p*-value (>0.05) indicated the desirable lack of fit. The predicted R^2^ value (0.9941) and adjusted R^2^ value were in close agreement (0.9976). Close agreement was also observed for the predicted and actual zone of inhibition data in Table 1.

All three model terms had a significant effect on the zone of inhibition as per the p-value in Table 5. Further, when the coefficient value of model terms A, B, and C in the quadratic equation (Equation (4)) indicated that a desirable signal-to-noise ratio was higher than 4, the suggested model had a value of 79.149, indicating the adequate signal. Therefore, this model could be used to navigate the design space.

The generated quadratic equation on the interactions of the three independent variables on the zone of inhibition of the formulations is shown in Equation (7).

Y3 = +10.83 − 0.4081*A + 6.10*B + 1.31*C + 0.0076*A^2^ − 0.0177*A*B − 0.1427*A*C − 0.8133*B^2^ + 0.3573*B*C + 0.5681*C^2^ (7)

**Figure 4:**
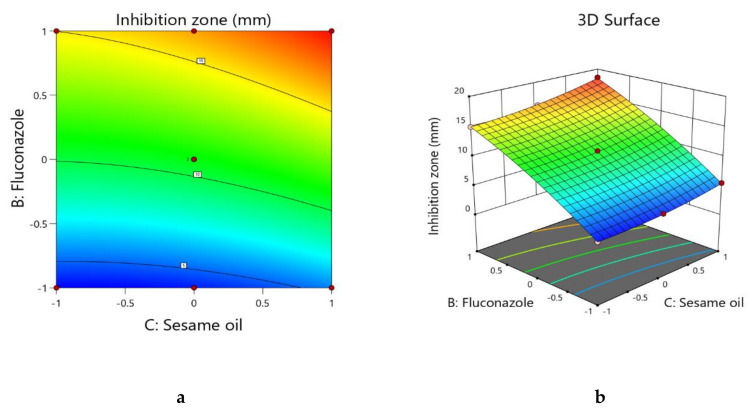
(**a**) Contour plot showing the interactions of sesame oil and fluconazole on the zone of inhibition of the FS-NTF. (**b**) Three-dimensional surface plot showing the interactions of sesame oil and fluconazole on the zone of inhibition of the FS-NTF.

Model term B had the highest coefficient (+6.10) as was expected because as the fluconazole concentration increased the zone of inhibition also increased. Although model terms A and C exhibited a significant effect as per the *p*-value in Table 5, model term B had the maximum effect on the zone of inhibition. For the coefficient values, model term A exhibited an inverse effect whereas model term C exhibited a proportional relationship on the zone of inhibition. Similar findings on the effects of models A, B, and C are evident in the contour plot and three-dimensional surface plot (Figure 4a,b). In the contour plot and three-dimensional surface plot, no color changes were observed through the sesame oil axis.

#### 3.2.4. Box–Behnken Statistical Designs: Optimization of the Ulcer Index of the Fluconazole‒-Sesame Oil Nanotransfersome Formulations

During optimization of the interactions of the three independent variables on the ulcer index of the FS-NTF, the linear model was found to be the best fit. The statistical outcome of the suggested linear model for the ulcer index is seen in Table 6. The model was found to be significant. Close agreement was observed in the predicted and actual ulcer indexes in Table 1 and in the adjusted R^2^ value (0.9771) and predicted R^2^ value (0.9671).

It could be said that model terms B and C had a significant effect, whereas the effect of model term A was insignificant, as per the *p*-value in Table 6. The generated linear equation for the interactions of the three independent variables with the ulcer index of the formulations is shown in Equation (8).

Y4 = +2.55 − 0.0166*A − 0.6701*B − 1.46*C(8)

**Figure 5:**
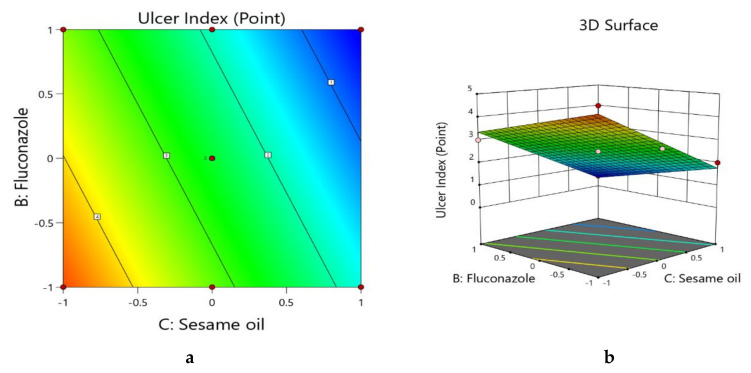
(**a**) Contour plot showing the interactions of the sesame oil and fluconazole on the ulcer index of the FS-NTF. (**b**) Three-dimensional surface plot showing the interactions of the sesame oil and fluconazole on the ulcer index of the FS-NTF.

The negative coefficient of significance for model terms B and C in Equation (8) represent a decrease in the ulcer index with an increasing amount of fluconazole and sesame oil, and this is in accordance with reported data [2,10]. Further, the maximum coefficient value of model term C represents a maximum effect of the sesame oil rather than the fluconazole, as per the contour plot and three-dimensional surface plot (Figure 5a,b), where the maximum effect of the sesame oil is reflected, followed by the amount of fluconazole.

### 3.3. Formulation of Optimized FS-NTF and FS-NTF-Loaded Hyaluronic Acid Hydrogel

Finally, the optimized nanotransfersome formulation was developed with the use of lecithin (level: −0.99 ≈ −1) 75 mg, fluconazole (level: 0.32 ≈ 0.3) 60 mg, and sesame oil (level: +0.99 ≈ 1) 75 mg. The predicted and observed values for the responses of the optimized formula are summarized in Table 7 as suggested by the software based on an analysis of the actual and predicted data. Values of the expected and adjusted R^2^ of the studied responses were in close agreement, proving the significance and predictive capacity of the design. Moreover, the experimental and predicted ratios with a percentage error of less than 10% and acceptable residuals were found between the real and predicted responses, showing a lack of curvature in the responses and the validity of the model (Table 5).

Hyaluronic acid, a natural biomolecule, has shown its antimicrobial effectiveness against bacteria and fungi in a dose-dependent manner [30]. Another contemporary study revealed a fungistatic role of hyaluronic acid through its inhibitory effect on the fungal lysozyme and peroxidase system [11]. Further, hydrogel containing hyaluronic acid has an effective and safe coating, facilitating the proliferation of basal keratinocytes and promoting reepithelialization. This role of hyaluronic acid controls the hydration of ulcer tissues and also the inflammatory process [31]. Therefore, the hyaluronic acid hydrogel was formulated in the present study of the formulated FS-NTF and was incorporated to facilitate the application of the developed formulation at the oral mucosa.

### 3.4. Rheological Evaluation of the HA-FS-NTF Hydrogel

The naturally available polysaccharide hyaluronic acid has been explored for incorporation in several pharmaceutical preparations because of its unique viscoelastic properties and effective augmentation of soft tissues. However, the degree of cross-linking and preparation method, along with the molecular weight of the natural polymer, have been shown to have a direct relationship on the rheological properties of the final product [32]. Therefore, the impact of our method of preparation and the cross-linking with Gantrez^®^ S-97 on the rheological property of the formulated hydrogel of hyaluronic acid was evaluated in the present study through an analysis of different parameters by the Brookfield viscometer.

**Figure 6:**
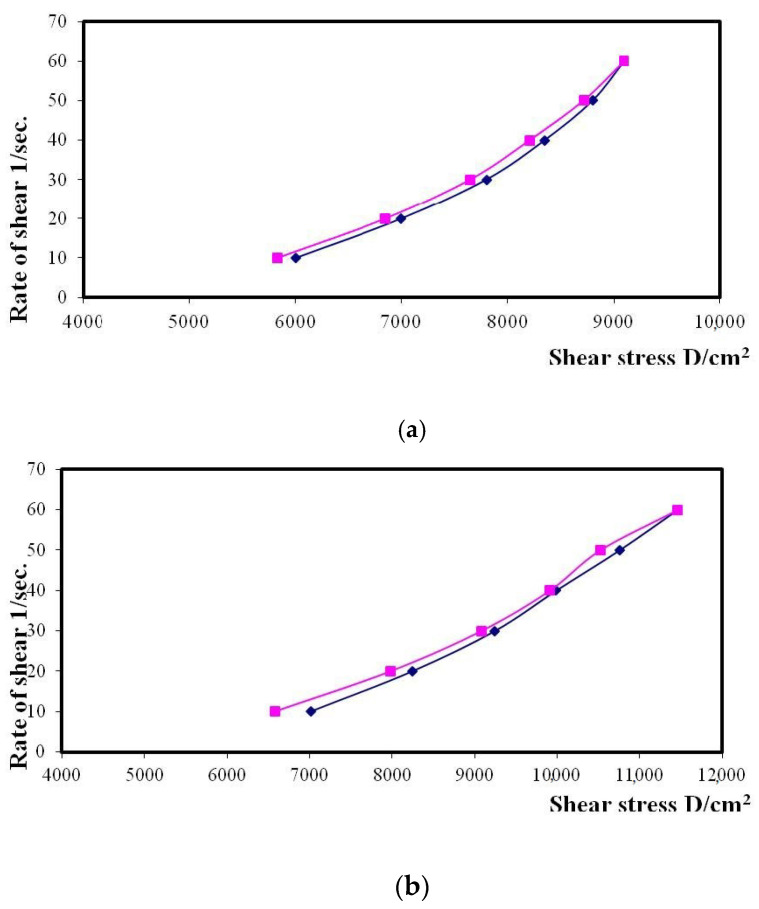
Rheogram of the (**a**) blank hyaluronic acid hydrogel and (**b**) HA-FS-NTF.

It is clear from Figure 6a that there was an obvious increase in the rate of shear with the increase in the shear stress of the hyaluronic acid hydrogel. Further, incorporation of the FS-NTF within the formulated hydrogel was found to be associated with an increase in the shear rate of the developed formulation (Figure 6b). The ηmin for the FS-NTF-loaded hyaluronic acid hydrogel was recorded as 132,318 cp, a number much higher than that for the ηmin for the blank hyaluronic acid hydrogel (110,016 cp). Therefore, application of this formulation within the oral cavity might promote in increasing shear rate during talking, eating, or smiling [33]. This increasing shear rate might promote longer retention of the formulation at the site of application for the effective healing of the ulcers.

**Figure 7:**
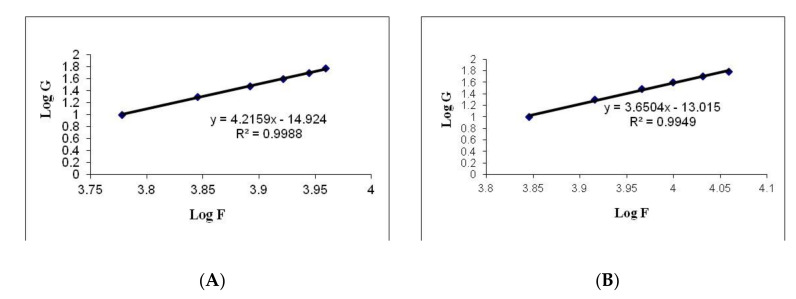
Representative plots of the logarithm of the rate of shear (G) versus the logarithm of the shearing stress (F) for (**A**) blank hyaluronic acid hydrogel and (**B**) HA-FS-NTF.

Values calculated by the Farrow’s constant (N) indicate a deviation in the fluid of the Newtonian flow behavior, where an increased value indicates a change in the flow behavior toward a more non-Newtonian flow [34]. Calculations for the values of the Farrow’s constant (N) were performed by plotting the logarithm of the shear rate (G) versus the logarithm of the shear stress (F), where the slope indicates the value of N (Figure 7). A value of less than 1 indicates dilatancy, and a value greater than 1 indicates pseudoplastic flow [34]. The flow behavior of the formulated hydrogels is presented in Table 8. All the hydrogel formulations had thixotropic behavior with pseudoplastic flow, and that is a desirable property for pharmaceutical gels for oral use. This special flow characteristic of hyaluronic acid hydrogel could be due to the breakdown of the structural configuration through the interruption of the existing intermolecular interactions between polymeric chains upon application of shear. There is further reoccurrence of polymerization between the molecules due to the force of the van der Waals interaction between the molecules upon removal of such shear [35,36]. The present study outcomes indicate that the optimal formulation of the HA-FS-NTF could facilitate the mean residence time within the oral cavity. This attribute is mostly due to the greater affinity of hyaluronic acid for the oral membrane due to its viscosity.

**Figure 8:**
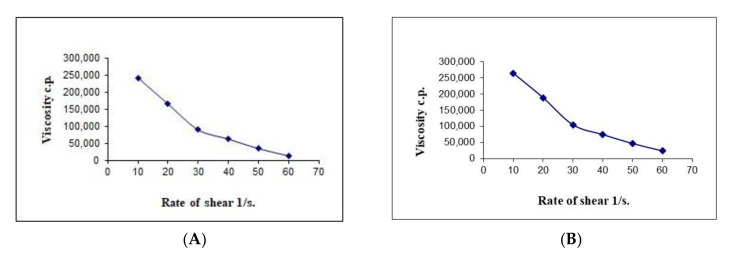
Representative plots of the rate of shear (G) versus the viscosity (η) for the (**A**) blank hyaluronic acid hydrogel and (**B**) HA-FS-NTF.

The results in Table 8 show that the viscosity of the developed formulations had different values at a minimum rate of shear (ηmin) and maximum rate of shear (ηmax); the values of the ηmax were less than those for the ηmin for both the formulations. Therefore, it could be assumed from our previous result that there might be structural breakdown of the polymeric chains during shear. Figure 8 shows the relation between the viscosity and shear rate of different gel bases at various concentrations.

We previously discussed the non-Newtonian behavior of the hyaluronic acid hydrogel. Figure 8 demonstrates that there is an inverse relationship between the viscosity of hydrogel and the applied shear rate. Therefore, the pseudoplastic flow of the formulated hydrogel is confirmed from the obtained results. Our results are in agreement with the available information in the literature on the relationship between shear rate and viscosity [36].

### 3.5. In Vitro Release Profile of the HA-FS-NTF

**Figure 9:**
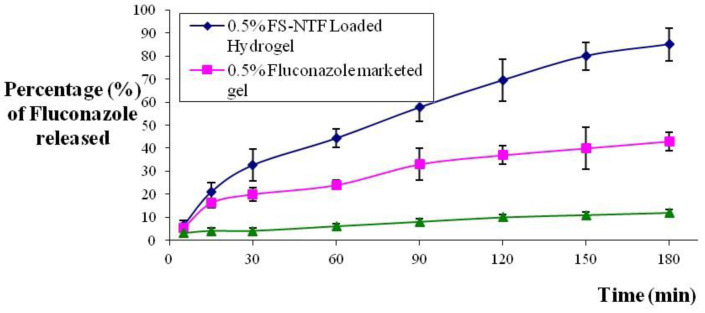
In vitro release profile of fluconazole from aqueous suspension, marketed gel, and HA-FS-NTF formulation. Data are expressed as mean ± SD (*n* = 3).

The results of the in vitro release study of the formulated HA-FS-NTF containing the 0.5% marketed formulation of fluconazole and the HA suspension containing the 0.5% fluconazole using the Type I USP dissolution apparatus are presented in Figure 9. From the results, it could be clearly seen that the release of fluconazole from the HA-FS-NTF was faster and completed within the time frame of 3 h, when compared with the other two formulations. The release of drug from the marketed gel and aqueous suspension was slow, at approximately 43% and 12%, respectively. There was a significantly higher rate of release (<0.05) for our formulated FS-NTF-loaded hydrogel (~85%). From Figure 9, it can be demonstrated that the initial release rate of the drug from the HA-FS-NTF was retarded due to incomplete gel formation, but the release of fluconazole was gradual following complete hydration of the gel and remained at a steady state thereafter owing to pseudoplastic flow. Swelling of the polymer due to hydration led to a change in the physical and chemical parameters of the hydrogel configuration, to tune the porous structure uniformly [37]. The decreased release of fluconazole from the suspension and even from the gel might be due to a larger particle size or a less porous configuration, whereas the development of the nanotransfersome delivery facilitated the release of the drug from the semipermeable membrane owing to the smaller vesicle size and enhanced solubility. Our results are in agreement with those of the existing literature, where the release of an entrapped hydrophobic agent (resveratrol) was found to be improved when delivered via this nanotransfersome method [26].

### 3.6. Permeation Parameters of Fluconazole from Optimized HA-FS-NTF by Ex Vivo Permeation Studies

The cumulative permeation of fluconazole through the sheep buccal mucosa was found to be 400 ± 57, 294 ± 34, and 122 ± 18 μg/cm^2^ (Table 9) after 3 h of an experimental period from the 0.5% HA-FS-NTF optimized hydrogel, marketed 0.5% fluconazole gel, and aqueous 0.5% fluconazole suspension, respectively. With respect to the examined formulations, the optimized HA-FS-NTF hydrogel had maximum fluconazole permeation across the oral mucosa, and this is statistically significant (*p* < 0.05) when compared with the other two formulations. Our ex vivo permeation results of the tested formulations could be correlated with the in vitro release profile of the respective formulations, where the highest in vitro release from the HA-FS-NTF is reflected by the highest ex vivo permeability from the same formulation (400 μg/cm^2^). The D, EF, Pc, and Jss of the optimized HA-FS-NTF were found to be superior when compared with those of the marketed gel and aqueous suspension (Table 9).

The maximum permeation of fluconazole through the buccal mucosa could be explained by the fact that the presence of nanosized vesicles containing the drug provided a platform for rapid skin permeation, which could be through transcellular and paracellular pathways [28,38]. Additionally, the presence of an edge activator in the HA-FS-NTF formulation (i.e., a surfactant, Tween 20^®^) might also act as a penetration enhancer that might aid in drug transport across the skin [39]. The presence of the edge activator in the HA-FS-NTF interchange with keratin-based filaments with a disruption is advantageous for drug transport. Oral thrush is not just a superficial manifestation, but the fungal growth also affects deeper skin layers. Therefore, permeation of fluconazole into deeper layers is of the utmost importance.

### 3.7. Zone of Inhibition against Candida Albicans

The antifungal efficacy using the disk diffusion technique was established to monitor the susceptibility of fluconazole in different laboratory settings in a cost-saving manner [40,41]. The accuracy and precision of the method could be comparable to broth microdilution minimum inhibitory concentration MIC testing [41].

**Figure 10:**
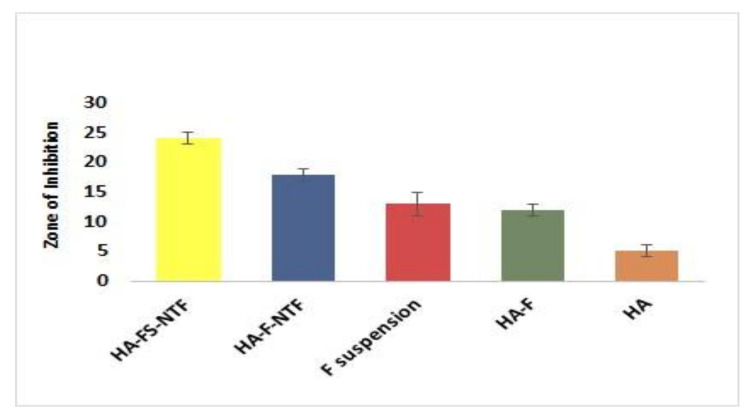
Representation of inhibitory zone diameters (mm) following 48 h of incubation of the agar plates containing *C. albicans*. Data are expressed as mean ± SD (*n* = 3).

The inhibitory zone diameters of the respective samples were measured at transitional points where the growth of *C. albicans* abruptly decreased. The areas were distinguished by a marked decrease in colony sizes. The diameters of the zone were measured after 48 h, and the readings are presented in Figure 10. The figure clearly shows that the inhibitory zone was highest for the HA-FS-NTF, and that the lack of sesame oil (HA-F-NTF) in the formulation was associated with a significant decrease in the zone of inhibition. This could be explained by the lack of antifungal properties of sesame oil in the formulation [10]. Alternatively, the zone of inhibition of the fluconazole suspension (F-Suspension) and hyaluronic acid containing fluconazole (HA-F) showed similar results; however, a comparatively greater diameter was observed with the latter one. This might be due to the additional effect of hyaluronic acid on the growth of *C. albicans* [11,30], which was also evident in the results of the blank hydrogel (HA) of hyaluronic acid (1.33 ± 0.58 mm).

#### Inhibitory Effect of Oral Candidiasis

Healthy rats were immunocompromised and tested for negative results of *Candida* in the oral cavity. Thereafter, confirmation of *Candida* infection was confirmed in the oral cavity of the immunocompromised animals. Daily treatment with five different formulations in the groups of animals with a sterile cotton swab revealed the improvement of oral infection as measured by the decrease in the ulcer index (Table 10). The results demonstrated that the HA-FS-NTF would be effective in controlling oral ulcers in the animals when compared with the other treatment options. The findings are quite similar to the in vitro inhibitory efficacy against *C. albicans*. Lack of sesame oil in the hyaluronic acid hydrogel loaded with F-NTF showed a marked difference in the ulcer index; however, the difference is statistically insignificant. Such a difference in efficacy in the HA-FS-NTF could be explained by the additional antifungal effect of sesame oil. Additionally, lack of hyaluronic acid in the fluconazole suspension was found be associated with a significant decrease in efficacy when compared with the hyaluronic acid hydrogel loaded with fluconazole; this could be illuminated by the additional role of hyaluronic acid in the control of *C. albicans* growth.

## 4. Conclusions

In summary, an optimal FS-NTF formulation was successfully developed by using the Design Experiment model, where the developed nanotransfersome was embedded into the hyaluronic acid hydrogel matrix. The in vitro drug release pattern and ex vivo permeation study through the sheep buccal mucosa of HA-FS-NTF showed a rapid release of the entrapped drug with significantly increased permeation parameters. These findings are positive indicators for local application in the oral cavity. The rheological properties of the HA-FS-NTF also support the localized application of the developed formulation. An in vitro antifungal assay and in vivo assessment of the ulcer index suggest a beneficial role for the developed formulation against oral candidiasis. The improved antifungal efficacy in our HA-FS-NTF might be due to the synergistic effect of hyaluronic acid, sesame oil, and fluconazole. Therefore, it can be summarized that fluconazole can effectively be delivered for the treatment of oral candidiasis when it is entrapped in a nanotransfersome carrier and embedded into cross-linked hyaluronic acid hydrogel.

## Figures and Tables

**Table 1:** Experimental runs in Box–Behnken statistical design: The independent variables and experimental dependent variables.

Run	Values of Independent Variables			Dependent Variable (Y)							
	A: Lecithin	B: Fluconazole	C: Sesame Oil	Globule Size (nm) (Actual)	Globule Size (nm) (Predicted)	EE (%) (Actual)	EE (%) (Predicted)	Inhibition Zone (mm) (Actual)	Inhibition Zone (mm) (Predicted)	Ulcer Index Score (Point) (Actual)	Ulcer Index Score (Point) (Predicted)
1	75	50	25	146.00	144.45	68.00	68.54	10.50	10.36	4.00	4.03
2	150	25	25	186.00	187.24	60.00	59.93	3.50	3.53	5.00	4.68
3	150	50	50	218.00	219.67	75.00	75.19	11.00	10.83	2.50	2.55
4	150	75	25	266.00	263.03	54.00	54.01	15.00	15.01	3.00	3.34
5	75	25	50	110.00	112.84	57.00	56.96	4.50	4.31	3.00	3.24
6	225	50	25	240.00	244.65	77.00	76.72	10.00	9.82	4.00	4.00
7	225	25	50	200.00	193.54	64.00	64.64	3.50	3.53	3.00	3.21
8	225	50	75	232.00	235.11	79.00	78.70	12.00	12.16	1.00	1.07
9	225	75	50	293.00	291.53	58.00	58.17	15.50	15.69	2.00	1.87
10	150	25	75	173.00	175.00	63.00	62.96	5.50	5.44	2.00	1.76
11	150	75	75	252.00	250.99	56.00	56.49	18.50	18.35	0.50	0.4190
12	75	50	75	134.00	129.72	72.00	72.08	13.00	13.26	1.00	1.11
13	150	50	50	221.00	219.67	76.00	75.19	10.50	10.83	2.50	2.55
14	75	75	50	160.00	166.64	52.00	51.05	16.50	16.55	2.00	1.90
15	75	25	25	120.00	118.63	55.00	54.79	3.50	3.78	5.00	4.70
16	75	50	50	141.00	138.72	70.00	70.58	11.50	11.24	2.50	2.57
17	150	25	50	181.00	182.75	62.00	61.71	4.00	3.91	3.00	3.22
18	150	75	50	260.00	258.64	55.00	55.52	16.00	16.11	2.00	1.88
19	225	75	75	285.00	285.18	59.00	58.76	18	17.79	0.5	0.4024

Dependent variables: Y1 = globule size of the prepared fluconazole-loaded sesame oil containing nanotransfersomes (FS-NTF); Y2 = Entrapment Efficiency Percentage (EE%) of fluconazole within the prepared FS-NTF; Y3 = zone of inhibition against *Candida albicans*; Y4 = ulcer index score.

**Table 2:** Scoring table of the oral ulcers created post-infection of *Candida albicans.*

Condition	Score
Normal-colored epithelial lining	0.5
Red coloration	1
Spot ulceration less than 1 mm	2
Ulcers between 1 and 2 mm without hemorrhagic streaks	2.5
Ulcers between 1 and 2 mm with hemorrhagic streaks	3
Ulcers > 2 mm but < 3 mm	4
Ulcers > 3 mm	5

The scores of the respective animals in each groups were recorded and analyzed.

**Table 3:** Statistical outcome (ANOVA) of the interaction of three independent variables on globule size of FS-NTF.

Source	Mean Square	F-Ratio	*p*-Value
Model	6429.44	321.44	<0.0001
A-Lecithin	26,799.61	1339.85	<0.0001
B-Fluconazole	16,346.65	817.25	<0.0001
C-Sesame oil	346.17	17.31	0.0024
AB	581.84	29.09	0.0004
AC	8.04	0.4020	0.5418
BC	0.0114	0.0006	0.9815
A^2^	3565.26	178.25	<0.0001
B^2^	4.02	0.2010	0.6645
C^2^	11.54	0.5770	0.4669
Residual	20.00		
Lack of Fit	21.94	4.88	0.3373
Pure Error	4.50		
Correlation Total			

**Table 4:** Statistical outcome (ANOVA) of the interactions of three independent variables on the EE% of the FS-NTF.

Source	Mean Square	*F*-Value	*p*-Value
Model	152.40	386.29	<0.0001
A-Lecithin	138.98	352.26	<0.0001
B-Fluconazole	108.87	275.96	<0.0001
C-Sesame oil	17.88	45.32	<0.0001
AB	0.0908	0.2302	0.6428
AC	0.7177	1.82	0.2104
BC	0.0908	0.2302	0.6428
A^2^	3.37	8.55	0.0169
B^2^	1057.67	2680.86	<0.0001
C^2^	0.3050	0.7730	0.4021
Residual	0.3945		
Lack of Fit	0.3813	0.7627	0.7147
Pure Error	0.5000		
Correlation Total			

**Table 5:** Statistical outcome (ANOVA) of the interactions of three independent variables on the zone of inhibition of the FS-NTF.

Source	Mean Square	*F*-Value	*p*-Value
Model	54.55	818.62	<0.0001
A-Lecithin	1.69	25.35	0.0007
B-Fluconazole	422.36	6338.58	<0.0001
C-Sesame oil	16.17	242.74	0.0817
AB	0.0015	0.0223	0.8845
AC	0.0970	1.46	0.2583
BC	0.6086	9.13	0.0144
A^2^	0.0002	0.0036	0.9536
B^2^	2.55	38.23	0.0002
C^2^	1.40	21.02	0.3413
Residual	0.0666		
Lack of Fit	0.0593	0.4747	0.8153
Pure Error	0.1250		

**Table 6:** Statistical outcome (ANOVA) for the linear model showing the interactions between the three independent variables and the ulcer index of the FS-NTF.

Source	Mean Square	*F*-Value	*p*-Value
Model	10.28	257.20	<0.0001
A-Lecithin	0.0028	0.0712	0.7932
B-Fluconazole	5.10	127.57	<0.0001
C-Sesame oil	20.12	503.27	<0.0001
Residual	0.0400		
Lack of Fit	0.0428		
Pure Error	0.0000		

**Table 7:** Composition of the actual and predicted responses of the optimal FS-NTF formulation.

Factor	Optimal Value	Response Variable	Actual Value	Predicted Value
A: Lecithin (mg)	75	Globule size of the prepared FS-NTF	140	138.6
B: Fluconazole (mg)	60	EE% of fluconazole within the prepared FS-NTF	70	69.2
C: Sesame oil (mg)	75	Zone of inhibition against *Candida albicans*	14.5	15.3
		Ulcer index score	1	0.88

**Table 8:** Rheological parameters of blank hyaluronic acid hydrogel and HA-FS-NTF.

Formulations	Viscosity * (Minimum) (cP)	Viscosity * (Maximum) (cP)	Farrow’s Constant (N)	Flow Behavior
Blank hyaluronic acid hydrogel	110,016 ± 7812	1535 ± 206	3.0111	Pseudoplastic
HA-FS-NTF	132,318 ± 9513	1907 ± 275	3.0024	Pseudoplastic

* Data are expressed as mean ± SD (*n* = 3).

**Table 9:** Parameters of permeation for fluconazole across the buccal mucosa for different formulations.

Parameters of Permeation	Optimized HA-FS-NTF Formulation	Fluconazole 0.5% Marketed Gel	Fluconazole Aqueous Suspension (0.5%)
Cumulative amount permeated (μg/cm^2^)	400 ± 57	294 ± 34	122 ± 18
Steady state flux, Jss, (μg/cm^2^.min)	2.223	1.633	0.677
Permeability coefficient, Pc, (cm/min)	4.1 × 10^−5^	3.2 × 10^−5^	1.3 × 10^−5^
Diffusion coefficient, D, (cm^2^/min)	13.12 × 10^−5^	8.11 × 10^−5^	1.53 × 10^−5^
Relative permeation rate (RPR)	1.36	-	0.414
Enhancement factor (EF)	3.278	2.409	-

**Table 10:** In vivo ulcer index of the *Candida* albicans‒infected immunocompromised animals after 3 days of treatment.

Tested Formula	Ulcer Index
HA-FS-NTF	0.67 ± 0.29
Hyaluronic acid hydrogel loaded with F-NTF (without sesame oil)	1.33 ± 0.28
Hyaluronic acid hydrogel loaded with fluconazole	2.17 ± 0.29
Fluconazole aqueous dispersion	2.83 ± 0.29
Blank hyaluronic acid hydrogel	4.67 ± 0.38

Data are expressed as mean ± SD (*n* = 3).

## Data Availability

Not applicable.
